# Factors Contributing to Residual Low Back Pain after Osteoporotic Vertebral Fractures

**DOI:** 10.3390/jcm11061566

**Published:** 2022-03-12

**Authors:** Hiroyuki Inose, Tsuyoshi Kato, Shoichi Ichimura, Hiroaki Nakamura, Masatoshi Hoshino, Shinji Takahashi, Daisuke Togawa, Toru Hirano, Yasuaki Tokuhashi, Tetsuro Ohba, Hirotaka Haro, Takashi Tsuji, Kimiaki Sato, Yutaka Sasao, Masahiko Takahata, Koji Otani, Suketaka Momoshima, Takashi Hirai, Toshitaka Yoshii, Atsushi Okawa

**Affiliations:** 1 Department of Orthopaedic and Trauma Research, Graduate School, Tokyo Medical and Dental University, Tokyo 108-0075, Japan; 2 Department of Orthopaedics, Ome Municipal General Hospital, Tokyo 198-0042, Japan; katoorth@gmail.com; 3 Department of Orthopaedics, Graduate School, Tokyo Medical and Dental University, Tokyo 108-0075, Japan; hirai.orth@tmd.ac.jp (T.H.); yoshii.orth@tmd.ac.jp (T.Y.); okawa.orth@tmd.ac.jp (A.O.); 4 Department of Orthopaedics, Kyorin University, Tokyo 181-8611, Japan; ichimura@ks.kyorin-u.ac.jp; 5 Department of Orthopedic Surgery, Graduate School of Medicine, Osaka City University, Osaka 545-8585, Japan; hnakamura@med.osaka-cu.ac.jp (H.N.); hoshino717@gmail.com (M.H.); shinji@med.osaka-cu.ac.jp (S.T.); 6 Department of Orthopedic Surgery, Osaka City General Hospital, Osaka 534-0021, Japan; 7 Department of Orthopaedic Surgery, Hamamatsu University of Medicine, Shizuoka 431-3192, Japan; togawa@med.kindai.ac.jp; 8 Departments of Orthopaedics and Rheumatology, Kinki University Nara Hospital, Nara 630-0293, Japan; 9 Department of Orthopedic Surgery, Niigata University Medical and Dental Hospital, Niigata 951-8520, Japan; thirano@med.niigata-u.ac.jp; 10 Department of Orthopaedic Surgery, Nihon University, Tokyo 173-8610, Japan; maruto10@gmail.com; 11 Department of Orthopaedic Surgery, University of Yamanashi, Yamanashi 409-3898, Japan; tooba@yamanashi.ac.jp (T.O.); haro@yamanashi.ac.jp (H.H.); 12 Department of Orthopaedic Surgery, Kitasato University Kitasato Institute Hospital, Tokyo 108-8642, Japan; tsuji9@gmail.com; 13 Department of Orthopaedic Surgery, Kurume University School of Medicine, Kurume University, Fukuoka 830-0011, Japan; kimiaki@med.kurume-u.ac.jp; 14 Department of Orthopaedic Surgery, Graduate School, School of Medicine, St. Marianna University, Kanagawa 216-8511, Japan; sasaospine@marianna-u.ac.jp; 15 Department of Orthopaedic Surgery, Hokkaido University Graduate School of Medicine, Hokkaido 060-8638, Japan; takamasa@med.hokudai.ac.jp; 16 Department of Orthopaedic Surgery, Fukushima Medical University School of Medicine, Fukushima 960-1295, Japan; kotani@fmu.ac.jp; 17 Department of Diagnostic Radiology, Center for Preventive Medicine, Keio University School of Medicine, Tokyo 160-8582, Japan; momo@rad.med.keio.ac.jp

**Keywords:** residual low back pain, osteoporotic vertebral fractures, nonunion, vertebral deformity, thoracolumbar alignment, radiographic evaluation, Cobb angle, alignment, visual analog scale

## Abstract

Although osteoporotic vertebral fractures (OVFs) are the most common type of osteoporotic fracture, few reports have investigated the factors contributing to residual low back pain in the chronic phase after OVFs by using radiographic evaluation. We examined the contribution of nonunion, vertebral deformity, and thoracolumbar alignment to the severity of residual low back pain post-OVF. This post hoc analysis of a prospective randomized study included 195 patients with a 48-week follow-up period. We investigated the associations between radiographic variables with the visual analog scale (VAS) scores for low back pain at 48 weeks post-OVF using a multiple linear regression model. Univariate analysis revealed that analgesic use, the local angle on magnetic resonance imaging, anterior vertebral body compression percentage on X-ray, and nonunion showed a significant association with VAS scores for low back pain. Multiple regression analysis produced the following equation: VAS for low back pain at 48 weeks = 15.49 + 0.29 × VAS for low back pain at 0 weeks + (with analgesics: +8.84, without analgesics: −8.84) + (union: −5.72, nonunion: −5.72). Among local alignment, thoracolumbar alignment, and nonunion, nonunion independently contributed to residual low back pain at 48 weeks post-OVF. A treatment strategy that reduces the occurrence of nonunion is desirable.

## 1. Introduction

The incidence of osteoporotic vertebral fractures (OVFs) increases gradually with age in populations aged >50 years [1,2]. As society ages, the number of patients with OVFs is expected to increase. In the acute phase after OVF, patients experience severe low back pain and deterioration in their quality of life [3]. Although most vertebral fractures improve with time, some patients continue to have low back pain even after the acute phase, and their quality of life continues to decline [4,5]. Therefore, residual low back pain after OVFs is becoming a major social problem with the advent of an aging society.

Various factors have been considered to be involved in the persistence of low back pain after OVF, including nonunion, deterioration of local spinal alignment, and deterioration of thoracolumbar alignment [6]. A recent cohort study of 67 patients reported that local deformity was associated with low back pain at 24 weeks after fracture [7]. However, it remains unclear which of these factors most contribute to the persistence of low back pain beyond 24 weeks after OVF. Accordingly, current evidence is limited because few reports have examined in detail the risk factors for residual pain in the chronic phase after OVFs using detailed radiographic evaluation. If we can identify the most important factors that contribute to residual low back pain after OVF development, we may be able to intervene therapeutically with those factors immediately after OVF development to prevent residual low back pain. This intervention strategy may improve the outcome of OVF treatment. Therefore, the purpose of this study was to determine the contribution of nonunion, vertebral deformity, and thoracolumbar alignment to the severity of residual low back pain after OVF.

## 2. Materials and Methods

This study is a post hoc analysis of a previous prospective randomized study (UMIN000014876) that compared the effectiveness of rigid and soft braces for acute thoracolumbar OVFs [8]. The original study enrolled 284 patients aged 65–85 years diagnosed with acute (within 4 weeks before inclusion) OVF between T10 and L2. Of these patients, 141 were randomly assigned to wear rigid braces and 143 to wear soft braces. Those in the rigid-brace group wore a rigid thoracolumbosacral brace, while those in the soft-brace group wore a soft thoracolumbosacral brace. Patients in both groups were instructed to wear braces at all times whenever possible during the 12-week period. The original study was approved by each hospital’s institutional review board, and prior to enrollment in the randomized controlled trial, all participants provided oral and written informed consent. The study’s detailed inclusion and exclusion criteria were previously described [8].

Patients with single-level acute thoracolumbar OVF who had received either rigid or soft brace treatment and had undergone lateral radiography at 0, 12, and 48 weeks and magnetic resonance imaging (MRI) at 48 weeks were included in the current study. Patients who could not be followed-up through to week 48 and those with incomplete imaging studies were eliminated from this study. The present study was approved by the institutional review board at the Tokyo Medical and Dental University and was compliant with the Declaration of Helsinki.

### 2.1. Patient-Reported Outcome Measure

The visual analog scale (VAS) scores for low-back pain (range, 0–100 points; higher scores indicate more severe pain) at 0 and 48 weeks after OVF were used [9]. In the original trial, the VAS scores were determined when the patients visited the hospital at 0, 12, and 48 weeks.

### 2.2. Radiographic Assessment

As radiological parameters, we measured the anterior vertebral body compression percentage on a lateral standing radiograph, as well as the local angle, T10/L2 Cobb angle, T10/L5 Cobb angle, lumbar lordosis, and sacral slope on a sagittal MRI at 48 weeks after OVF.

The ratio between the vertical height of the compressed anterior section of the injured vertebral body and the posterior vertebral body height at the same level was defined as the anterior vertebral body compression percentage. The angle formed by crossing lines drawn from the superior and inferior endplates of the fractured vertebrae was termed as the local angle. The T10/L2 Cobb angle was defined as the angle formed by intersecting lines drawn from the superior endplate of T10 and the inferior endplate of L2. The T10/L5 Cobb angle was defined as the angle formed by intersecting lines drawn from the superior endplate of T10 and the inferior endplate of L5. The angle between the superior endplate of L1 and the inferior endplate of L5 was defined as lumbar lordosis. The sacral slope was defined as the angle between the tangent to the upper endplate of S1 and the horizontal plane.

In this study, a subsequent vertebral fracture was defined as a fracture occurring within 48 weeks of an initial fracture at a different site. During the 48-week follow-up period, a new vertebral fracture was defined as a decrease of at least 20% in the height of any vertebral body. At the 48-week follow-up, nonunion was defined as a detectable gas- or fluid-filled cleft separating the superior and inferior endplates on MRI and radiography.

### 2.3. Data Analysis

We investigated the associations between background and 48-week variables with VAS scores for low back pain at 48 weeks in a univariate and multiple linear regression model. First, predictors associated with the dependent variable at a *p*-value ≤ 0.25 in univariate regression analyses were carried forward to the second step of the analysis [10]. Second, together with the baseline equivalent (VAS score at week 0) of the dependent variable, the remaining predictors were included in a backward multiple regression analysis. Including the baseline equivalent is a routine approach in prediction analysis, because this variable is frequently the most important predictor in the regression model [11]. Predictors having a *p*-value greater than 0.1 were eliminated. Then, odds ratios and their approximate 95% confidence intervals for residual low back pain were calculated. Finally, the forced-entry multiple regression analysis was performed using variables related to nonunion, local vertebral deformity, and thoracolumbar alignment, as well as variables selected for backward regression analysis. For all statistical analyses, JMP version 14 (SAS Institute, Cary, NC, USA) was used. All tests were two-sided, and *p*-values < 0.05 were considered significant.

## 3. Results

### 3.1. Demographics

**Figure 1:**
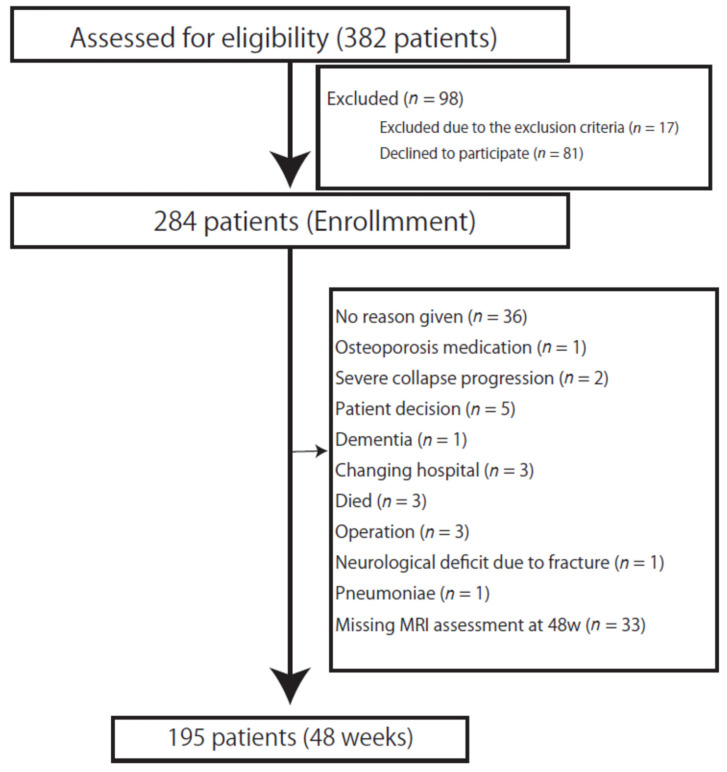
Study participant flow diagram. During the study period, 382 patients were assessed for eligibility. The original study enrolled 284 patients. During the follow-up period, an additional 89 patients were excluded. MRI, Magnetic resonance imaging.

In total, 195 patients with 48 weeks of follow-up were involved in this study. The exclusion criteria are shown in Figure 1. Table 1 shows the baseline characteristics of the patients.

### 3.2. Factors Contributing to Residual Low Back Pain

We investigated the association between the baseline and 48-week radiographic variables and VAS for low back pain at 48 weeks in the univariate regression analysis (Table 2). The univariate analysis revealed that VAS for low back pain at week 0, the use of analgesics, local angle on MRI, anterior vertebral body compression percentage on radiography, and nonunion had a significant association with the VAS score for low back pain at 48 weeks after OVF.

Then, we evaluated the independent predictors for the VAS score for low back pain at 48 weeks using a multiple regression analysis. Based on the univariate analysis, the dependent variable was the VAS score for low back pain at 48 weeks, while the independent variables were the fracture level, VAS score for low back pain at week 0, use of analgesics at 48 weeks, local angle, T10/L2 Cobb angle, anterior vertebral body compression percentage, nonunion, and secondary fracture. As a result, the VAS score for low back pain at week 0 (regression coefficient = 0.29, *p* < 0.0001), the use of analgesics (regression coefficient = 8.84, *p* = 0.0003), and nonunion (regression coefficient = 5.72, *p* = 0.01) were identified as the independent predictors (Table 3).

**Figure 2:**
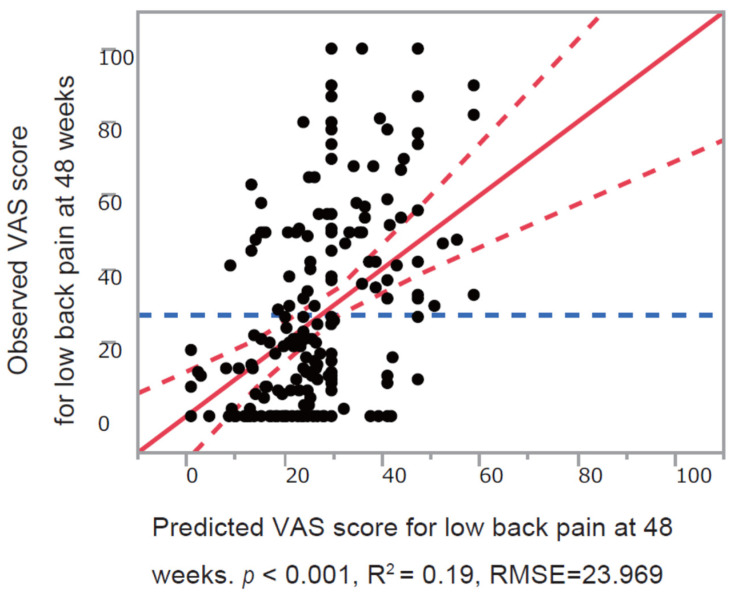
Observed versus predicted plots of multiple linear regression model for the VAS score for low back pain (48 weeks). The red line indicates regression line. The red dashed curve indicates a 0.05 level of significance. The blue dashed line indicates the mean VAS score for low back pain at 48 weeks. RMSE, Root mean squared error, VAS, visual analog scale.

According to this prediction model, the following equation was obtained (Figure 2):

(1) VAS for low back pain at 48 weeks =15.49+(union:−5.72, nonunion:5.72)+0.29× VAS for low back pain at 0 week +(using analgesics: 8.84, not using analgesics:−8.84),

The results show that patients with nonunion at 48 weeks had more low back pain than those with union. These results also show that patients with a severe low back pain in the acute phase were more likely to have residual low back pain, relative to those with a mild low back pain. 

Finally, we performed the forced-entry multiple regression analysis using variables related to nonunion, local vertebral deformity (anterior vertebral body compression percentage), and thoracolumbar alignment (T10/L2 Cobb angle), as well as variables selected for backward regression analysis. As a result, while nonunion was associated with VAS scores for low back pain at 48 weeks (*p* = 0.01), the anterior vertebral body compression percentage and the T10/L2 Cobb angle were not significantly associated with the VAS scores for low back pain at 48 weeks (*p* = 0.26 and 0.82, respectively) (Table 4).

## 4. Discussion

This study investigated the factors contributing to residual low back pain at 48 weeks after OVF. The univariate analysis showed that the use of analgesics, local angle on MRI, anterior vertebral body compression percentage on X-ray, and nonunion were associated with low back pain at 48 weeks. The multiple regression analysis showed that nonunion, the use of analgesics, and the VAS score for low back pain at week 0 were independent predictors of the VAS score for low back pain at 48 weeks after OVF. To our knowledge, this study is the first to show that nonunion contributed more to residual low back pain than other radiological factors, such as thoracolumbar alignment.

In this study, nonunion, local angle, and anterior vertebral body compression percentage showed a stronger association with the VAS score for low back pain compared to the T10/L5 Cobb angle and lumbar lordosis. In previous reports, vertebral body deformity and poor spinopelvic sagittal alignment were closely associated with chronic low back pain [12,13,14]. However, in patients with chronic low back pain after OVFs with coexisting local vertebral body deformity and spinopelvic malalignment, it remained unclear which treatment strategies should be considered to reduce low back pain. Based on the results of this study, considering therapeutic interventions to alleviate residual low back pain after thoracolumbar OVF, it can be interpreted that a better treatment strategy might be to focus on improving the local kyphosis angle, rather than on improving the T10/L5 Cobb angle. In fact, a retrospective study showed that vertebroplasty was effective in treating painful OVFs at 2–12 months after onset [15]. This treatment strategy should be tested in the future.

The multiple logistic regression analysis showed that nonunion was the independent factor that contributed to the residual low back pain 48 weeks after OVF. A recent cohort study of 67 patients found that the vertebral kyphosis angle at 24 weeks after OVF was significantly higher in the low back pain group, whereas the global sagittal alignment parameters showed no difference between the low back pain and non-low back pain groups [7]. In addition, they found that patients with nonunion showed significantly smaller vertebral kyphosis angles [7]. Therefore, the results of this study were consistent with those of the previous study; however, it is important to note that the previous study was an analysis of residual low back pain at 24 weeks after fracture. Collectively, it may be important to prevent the occurrence of nonunion to prevent residual low back pain after OVF.

At present, the indications for vertebroplasty in the acute phase are controversial [16,17,18]. In contrast, there are few reports that have investigated the usefulness of vertebroplasty for chronic fractures. In a prospective study comparing vertebroplasty and conservative treatment for painful OVFs more than 3 months after injury, the results of vertebroplasty were superior concerning pain improvement [19]. Furthermore, in a prospective randomized study of vertebral fractures with persistent pain for >3 months, vertebroplasty provided better pain control and improved quality of life than sham surgery, although the study is still in the preprint stage [20]. Accordingly, if nonunion or delayed union is observed in the chronic phase (>3 months) after OVF, it may be better to consider whether the pain can be alleviated with vertebroplasty rather than with thoracolumbar fusion surgery, irrespective of spinopelvic sagittal alignment. 

In addition, the incidence of nonunion 48 weeks after OVF was 17.5% [21]. If appropriate osteoporosis treatment can reduce the incidence of nonunion after OVF, it may improve the outcome of conservative treatment of OVF, as a retrospective study showed that the incidence of cleft formation was reduced in the teriparatide treatment group compared to the risedronate group [22]. Furthermore, the use of teriparatide also produced positive results for fracture healing in selected fracture types, such as atypical, periprosthetic, and atypical periprosthetic femoral fractures [23]. Naturally, further research is needed concerning surgery and osteoporosis medication.

This study had a limitation. Numerous patients were excluded after enrollment. Accordingly, attrition bias might limit the study’s internal validity. Second, local vertebral body deformity was assessed with upright X-rays, but global spinal parameters in the upright position were not assessed because MRI measurements in the sagittal plane were used. This was because the sagittal MRI had a wider imaging area than the lateral radiograph in this study and because it is desirable to have the same measurement conditions. In addition, intra- and inter-examiner reliability was better for MRI than for radiographs [24]. An observational study found that MRI can be used for estimating global lumbar lordosis [25]. Further studies are needed to solve these limitations and validate our findings.

## 5. Conclusions

In conclusion, this study demonstrated that among local alignment, thoracolumbar alignment, and nonunion, the latter was the independent factor contributing to residual low back pain at 48 weeks after OVF. Therefore, it is desirable to establish a treatment strategy that will reduce the occurrence of nonunion.

## Figures and Tables

**Table 1:** Patient demographics.

Characteristics	
Age, years (SD)	75.3 (5.3)
Fracture level, *n*	
T10	4
T11	13
T12	75
L1	67
L2	36
48 weeks variables	
Use of analgesics, *n* (%)	30 (15.4)
VAS for low back pain, point (SD)	27.5 (26.4)
Local angle, degree (SD)	−17.7 (6.3)
T10/L2 Cobb angle, degree (SD)	−16.1 (10.4)
T10/L5 Cobb angle, degree (SD)	18.6 (11.6)
Lumbar lordosis, degree (SD)	27.3 (13.4)
Sacral slope, degree (SD)	35.8 (8.0)
AVBCP, % (SD)	54.3 (16.4)
Nonunion, *n* (%)	35 (18)
Secondary fracture, *n* (%)	11 (6)

AVBCP, anterior vertebral body compression percentage; VAS, visual analog scale. SD, standard deviation.

**Table 2:** Univariate regression analysis. Association of baseline and 48 weeks variables with low back pain at 48 weeks after OVF.

Characteristic	B	95% CI	*p-*Value
Baseline variables			
Age	0.18	−0.54–0.89	0.63
Fracture level T12, L1, L2	−6.32	−12.89–0.26	0.06
VAS 0 week	0.34	0.20–0.48	<0.0001 *
48 weeks variables			
Use of analgesics	10.44	5.47–15.41	<0.0001 *
Local angle	−0.66	−1.24–−0.07	0.03 *
T10/L2 Cobb angle	−0.33	−0.69–0.03	0.08
T10/L5 Cobb angle	−0.12	−0.46–0.21	0.47
Lumbar lordosis	0.06	−0.23–0.34	0.70
Sacral slope	−0.07	−0.55–0.42	0.78
AVBCP	−0.30	−0.52–−0.07	0.01 *
Nonunion	6.69	1.91–11.48	0.01 *
Secondary fracture	5.79	−2.28–13.86	0.16

B, partial regression coefficient; AVBCP, anterior vertebral body compression percentage; VAS, visual analog scale; CI, confidence interval; * *p* < 0.05.

**Table 3:** Multiple regression analysis: independent factors contributing to low back pain at 48 weeks after OVF.

Factor	B	95% CI	*p-*Value
VAS at 0 week	0.29	0.15–0.42	<0.0001 *
Use of analgesics at 48 weeks	8.84	4.10–13.59	0.0003 *
Nonunion	5.72	1.30–10.15	0.01 *

B, partial regression coefficient; VAS, visual analog scale; OVF, osteoporotic vertebral fractures; CI, confidence interval. * *p* < 0.05.

**Table 4:** Forced-entry multiple regression analysis: independent factors contributing to low back pain at 48 weeks after OVF.

Factor	B	95% CI	*p-*Value
VAS at 0 week	0.29	0.15–0.43	<0.0001 *
Use of analgesics at 48 weeks	8.03	3.16–12.91	0.001 *
Nonunion	6.23	1.30–11.16	0.01 *
AVBCP	−0.13	−0.36–0.10	0.26
T10/L5 Cobb angle	−0.04	−0.35–0.27	0.82

B, partial regression coefficient; AVBCP, anterior vertebral body compression percentage; VAS, visual analog scale; CI, confidence interval. * *p* < 0.05.

## Data Availability

Not applicable.
